# Coercion promotes alloparental care in cooperative breeders

**DOI:** 10.1093/beheco/arac125

**Published:** 2023-02-28

**Authors:** Markus Zöttl, Tanja Schreier, Michael Taborsky

**Affiliations:** Division of Behavioural Ecology, Institute of Ecology and Evolution, University of Bern, CH-3032 Hinterkappelen, Switzerland; Department of Biology and Environmental Science, Linnaeus University, Kalmar, Sweden; Division of Behavioural Ecology, Institute of Ecology and Evolution, University of Bern, CH-3032 Hinterkappelen, Switzerland; Division of Behavioural Ecology, Institute of Ecology and Evolution, University of Bern, CH-3032 Hinterkappelen, Switzerland; Max Planck Institute of Animal Behavior, D-78467 Konstanz, Germany; Institute for Advanced Study (Wissenschaftskolleg) Berlin, D-14193 Berlin, Germany

**Keywords:** coercion, punishment, negotiation, cooperative breeding, pay-to-stay, *Neolamprologus pulcher*

## Abstract

Members of social groups may negotiate among each other about the exchange of goods and services. If this involves asymmetries between interacting partners, for instance in condition, power, or expected payoffs, coercion may be involved in the bargain. Cooperative breeders are excellent models to study such interactions, because asymmetries are inherent in the relationship between dominant breeders and subordinate helpers. Currently it is unclear whether punishment is used to enforce costly cooperation in such systems. Here we investigated experimentally in the cooperatively breeding cichlid *Neolamprologus pulcher* whether alloparental brood care provided by subordinates is contingent on enforcement by dominant breeders. We manipulated first the brood care behavior of a subordinate group member and then the possibility of the dominant breeders to punish idle helpers. When subordinates were prevented from providing brood care, breeders increased their attacks on them, which triggered increased alloparental brood care by helpers as soon as this was again possible. In contrast, when the possibility to punish helpers was prevented, energetically costly alloparental brood care did not increase. Our results confirm predictions of the pay-to-stay mechanism causing alloparental care in this species and they suggest more generally that coercion can play an important role in the control of cooperation.

## Teaser

When members of social groups negotiate about the exchange of goods and services, they may use coercion to increase the chances of a favorable outcome. However, it is still unclear whether animals use punishment to enforce cooperative behavior from others. In cooperatively breeding cichlids, we show that received aggression triggers increased alloparental brood care in subordinates. This suggests that fish punish idle helpers, such that these helpers then show increased helping effort.

## INTRODUCTION

Social interactions are typically characterized by a conflict of interest between agents differing in power (Hammerstein 1981; Rubinstein 1982; Binmore 2010; Phillips 2018; Taborsky et al. 2021). This asymmetry affects negotiations between social partners about access to resources, group membership, or other advantages (McNamara et al. 1999; Melis et al. 2009). Negotiation processes, the interaction by which individuals adjust their behavior to their partner’s behavior and signaling to achieve a beneficial outcome, may encompass combinations of reciprocal exchanges and elements of coercion, such as punishment for cheating or withheld cooperation (Clutton-Brock and Parker 1995; Raihani et al. 2012; Quinones et al. 2016; Taborsky et al. 2021). Currently, it is unclear to which extent coercion and enforcement affect mutual relationships and reciprocal exchanges in highly social animals.

In cooperative breeders, dominant individuals benefit from alloparental brood care performed by subordinate group members (Russell and Rowley 1988; Emlen and Wrege 1988; Brouwer et al. 2005; Zöttl et al. 2013b). In return, the latter often gain substantial direct fitness benefits from their group membership despite being reproductively suppressed (Taborsky 1984; Balshine-Earn et al. 1998; Kingma et al. 2011; Leadbeater et al. 2011). They enjoy access to the cooperatively defended territory with resources such as food and shelters and may benefit from enhanced safety from predators (Heg et al. 2004; Groenewoud et al. 2016). Later in life, they may inherit the breeding position in their natal group and benefit from the presence of helpers that previously they had helped to raise by alloparental care (Stiver et al. 2006, Kingma et al. 2011, 2014, Leadbeater et al. 2011). However, the presence of subordinate group members can impose costs to dominants through resource competition and by enhancing intra-sexual competition for reproduction (Webster et al. 2004; Mitchell et al. 2009a, 2009b; Hellmann et al. 2015). If these costs outweigh the benefits, the dominant individuals will not tolerate subordinates as group members and eventually evict them from their territory (Dierkes et al. 1999; Taborsky 1985, 2016; Zöttl et al. 2013c; Thompson et al. 2016).

Theoretical models suggest that subordinates may provide help to compensate for the costs imposed to dominants, thereby removing the incentive for forcible eviction (Gaston 1978; Kokko et al. 2002; Hamilton and Taborsky 2005). Such services provided in exchange of tolerance in the group have been referred to as “pay-to-stay” (PTS), which can lead to evolutionarily stable group living and cooperation (Quinones et al. 2016). This mechanism may involve a negotiation process and is particularly important when relatedness between dominants and subordinates is low, the presence of subordinates inflicts costs on dominants, dispersal and independent breeding by subordinates is constrained, and individuals are generally long-lived. The crucial difference from other mechanisms selecting for cooperative brood care, such as kin selection and benefits from group augmentation (García-Ruiz et al. 2022), is that subordinates do not provide help voluntarily but are forced by dominants to do so (Fischer et al. 2014; Naef and Taborsky 2020a, 2020b). PTS predicts that when subordinates fail to produce sufficient benefits to the dominants they are punished and eventually evicted from the territory (Gaston 1978; Mulder and Langmore 1993). However, the predicted causal effect of punishment on increased helping behavior has hitherto not been demonstrated in non-human animals (Raihani et al. 2012).

In the African cichlid fish *Neolamplologus pulcher* several key aspects of the mechanism regulating the asymmetric negotiation between dominants and subordinates that characterizes the pay-to-stay relationship have been experimentally demonstrated (Taborsky 1985; Balshine-Earn et al. 1998; Bergmüller and Taborsky 2005; Bergmuller et al. 2005; Heg and Taborsky 2010; Zöttl et al. 2013a, 2013d; Fischer et al. 2014; Naef and Taborsky 2020a, 2020b). For instance, individuals prevented from defending or subordinates that are temporarily removed from the group are subsequently attacked by more dominant helpers and breeders (Balshine-Earn et al. 1998; Fischer et al. 2014; Naef and Taborsky 2020a). Helpers prevented from defending the territory against intruders subsequently show increased levels of defense (Bergmuller et al. 2005; Naef and Taborsky 2020b), and a similar pattern emerged when temporarily preventing helpers from providing alloparental care (Zöttl et al. 2013b). Subsequent to the experimental manipulation of defense, helpers increased either cooperative defense or submissive behavior, suggesting that cooperative defense serves an appeasement function (Bergmuller and Taborsky, 2005). Similar results were obtained when helpers were experimentally prevented from digging out the territory shelter (Naef and Taborsky 2020a). In addition, subordinates are only accepted in the territory when help is needed (Taborsky 1985; Zöttl et al. 2013c). However, all these experiments manipulated the behavior or presence of subordinate helpers rather than the behavior of dominant breeders, so that the potentially underlying negotiation process between both parties is still poorly understood.

Here we tested experimentally whether breeders force non-breeders to provide alloparental care by direct physical attacks and very close proximity, which may signal the threat of imminent aggression in *N. pulcher*. First, we created a situation in which helpers either showed normal levels of alloparental brood care or were prevented to care for the dominant breeders’ clutch. Then we either prevented physical social interaction between breeders and helpers to inhibit the possibility of punishment, or we allowed unrestricted physical contact. We predicted that if physical social interaction between breeders and helpers is required to enforce help, more help should be provided in the treatment where social interaction is possible. In contrast, if the mere presence of dominant breeders suffices to induce help, increased levels of help should be observed after experimentally imposed idleness, regardless of whether physical interactions were possible of not.

## METHODS

### Experimental subjects and group formation

**Figure 1:**
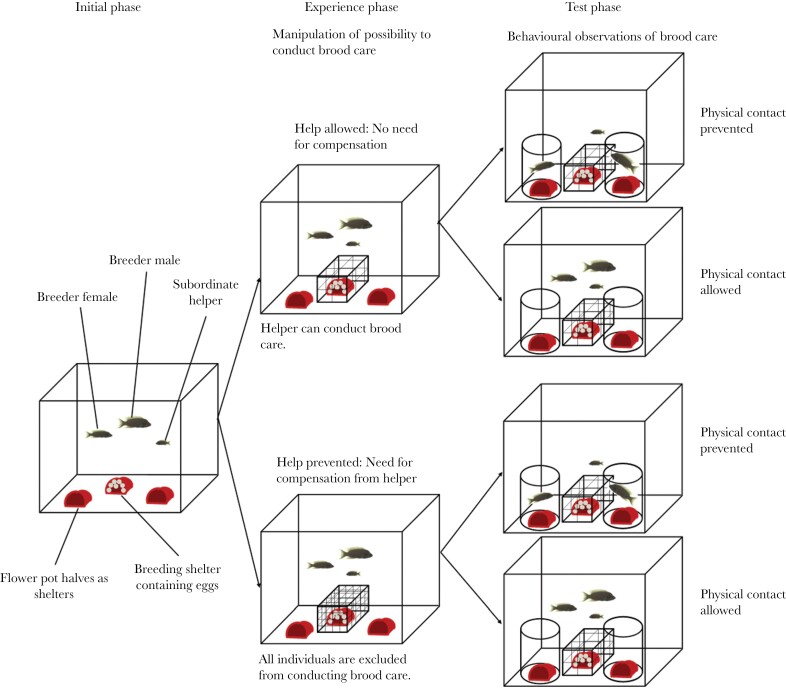
Sketch of the experimental set-up. The experiment started one or two days after the dominant female had spawned for the first time. In the “Initial phase”, fish behavior was recorded without experimental manipulation. In the experience phase, for 30 min either all group members were prevented from entering the breeding shelter (impassable mesh cage put over the breeding shelter; i.e., experience treatment “help prevented”), or the helper could access the breeding shelter to care for the eggs (mesh cage was passable for the helper; i.e., experience treatment “help allowed”). In the test phase, for 20 min the treatments either allowed physical contact between the breeders and helpers enabling punishment by physical attacks (ramming or biting; i.e., experimental treatment “physical contact allowed”), or the breeders were confined in clear perspex cylinders preventing bodily contact (i.e., experimental treatment “physical contact prevented”). All groups underwent all four possible combinations of experience and test phases in random order yielding a within subject, full factorial experimental design.

Experimental subjects were taken from the laboratory stock population originating from wild *N. pulcher* caught in Lake Tanganyika near Mpulungu, Zambia, in the years 1999, 2006, and 2009. The experimental groups were assembled following a standard procedure and consisted of a male, a female, and a subordinate helper of unknown sex (Taborsky 1984). Resembling the natural group composition, males were the largest individuals in the group (standard length (SL), mean ± SD: 63.8 mm ± 8.3) followed by females (55.8 mm ± 5.5) and helpers (26.6 mm ± 3.0). The minimal size difference between the male and female pair members was 0.5 mm, whereas the female was at least 10 mm larger than the respective helper. Each group was kept in a 100 L compartment of a subdivided 200 L aquarium (*N* = 17). All compartments contained a semi-transparent tube suspended below the water surface serving as a retreat shelter, an air-driven biological filter suspended in the water, and 4 flowerpot-halves serving as breeding shelters (see Figure 1). The bottom of the tank was covered with a mixture of gravel and sand. The water temperature was held constant at 26–28°C and the light regime was set at a 13 h-light:11 h-dark cycle, simulating natural conditions in Lake Tanganyika. The fish were fed six times a week with dried food and 2–4 times weekly with additional krill. The experiments were conducted at the Ethologische Station Hasli of the University of Bern, Switzerland between June and October 2012 under the ethical approval license of Veterinaeramt Bern 16/09.

In the first days after group formation, the fish were habituated to a mesh cage (mesh size 10 × 10 mm) that later in the experimental procedure was used to prevent breeders from entering the breeding shelter. Helpers learned that they could enter a shelter despite the mesh net put over it, whereas the breeders were unable to pass the mesh. After this initial habituation, families were checked for clutches every day. If eggs were found, the experiments took place on the first and second day after spawning.

The helpers habituated quickly to the introduced mesh cage. All helpers passed the mesh cage within a few days and our observations confirmed that all helpers had learnt the task before the experiment started. During the time between training and breeding the groups were exposed to the mesh net cage intermittently to ensure that they remained habituated to the experimental procedures.

### Experimental design and procedure

We started the experiment when the group produced their first clutch (Figure 1). At that point the fish were habituated to the experimental setup and the subordinates had learned that they could pass freely through the mesh net temporarily covering the breeding shelter (Supplementary Figure 1 and Supplementary Table 1). After a baseline behavior recording (“initial phase”), the group was exposed to one of two treatments in the experience phase. In the experience treatment “help prevented” the whole group was prevented from caring for the brood for 30 min by placing a close-meshed cage over the breeding shelter so that no fish could pass. Previous experiments have shown that individuals react to time periods where they were unable to conduct brood care by increasing their brood care investment subsequently (Zottl et al 2013d). In the experience treatment “help allowed” a broad-meshed cage was put over the breeding shelter for 30 min that allowed the helper to pass, but the breeders were excluded from entering the breeding shelter to provide egg care.

In the test phase immediately following the experience phase, each group was assigned to a treatment either preventing social interaction between breeders and helpers (“physical contact prevented”) or allowing social interaction (“physical contact allowed”, see Figure 1). In both situations, the broad-meshed cage was put over the breeding shelter which allowed helpers to care for the eggs whereas the breeders were excluded from entering the shelter. In the experimental treatment “physical contact prevented” physical interactions were prevented by restricting the breeders to an area in the tank using transparent perspex cylinders. Hence, they remained visually present and could interact with the helper, but they were unable to exert any overt physical aggression. In the “physical contact allowed” condition the cylinders were inserted in the same way, but the breeders remained outside and were unconstrained in their ability to physically attack helpers (Figure 1). All experimental groups underwent all treatments in the experience and test phases in random order, so that each group was tested four times. The minimum time between two consecutive trials was 1 hour. In total, 17 groups were used in the experiment and a total of 68 trials of the test phase were conducted. In 11 of these 17 groups, the experimental trials were conducted on two consecutive days, with two trials per day separated by at least an interval of one hour. In 6 groups we conducted all 4 trials on the same day, again with one hour minimum time between consecutive trials.

### Brood care and behavioral observations

In the test phase, we conducted continuous focal observation of 15 min on the helper individual (Altmann 1974) focusing on two alloparental care behaviors that have contrasting energetic implications. We recorded the time individuals engaged in fanning. This behavior serves to create a water current around the clutch and increases the supply of oxygen for the clutch. It is energetically costly because it involves a forward swimming movement that is compensated by intense beats with the pectoral fins to keep the fish on the spot (Grantner and Taborsky 1998; Taborsky and Grantner 1998). Second, we quantified egg cleaning by counting the frequency of micro nips towards the eggs, which serves a hygienic function by removing fungi and other microorganisms. This brood care behavior probably involves less energy expenditure (Taborsky and Grantner 1998). Additionally, in each phase of the experiment, we recorded social interactions between breeders and helpers during the 15 min focal observations. We present frequencies of overt physical aggression from both breeders towards the helper (ramming and biting), frequencies of restrained aggression (threat signals without physical contact), and the frequencies of submissive behavior (tail quivering) of the helper towards the breeders. We did not record other submissive behaviors (submissive postures and hook swimming) because they are usually shown less frequently and it is unclear whether they involve energetic costs comparable to tail quivering.

### Statistical analyses

To model fanning duration, we fitted the log transformed duration of fanning behavior as response variable and added the experimental condition of the experience phase (factor with two levels: help prevented/allowed) and the current experimental treatment (factor with two levels: physical interactions prevented/allowed) as fixed factors. We included the interaction between these fixed factors and specified a random effect that identified the experimental group. We modeled the data specifying a Gaussian distribution using a Linear Mixed Effect Model (LMM). To model egg cleaning behavior we fitted the number of egg cleaning events as the response variable and again added the experimental condition of the experience phase and the current experimental treatment as fixed factors. We included the interaction between the fixed factors and the identity of the test group as random effect. Because the egg cleaning count data showed signs of overdispersion we modeled the data specifying a negative binomial distribution using a Generalized Linear Mixed Effect Model (GLMM).

To answer the question whether breeders increased aggression towards the subordinate and whether the subordinate increased submission to the breeders in response to preventing the possibility to provide brood care we used the behavioral data collected in the experience phase and specified the frequency of overt aggression, restrained aggression and of submission as response variable in separate models. We fitted the treatment as factor with 2 levels (help allowed/ help prevented) and the group identity as random factor. We also modeled the distribution of aggressive and submissive behavior longitudinally throughout the experiment when the groups moved from the initial phase to the experience phase (help prevented) and then to the test phase (contact allowed) by fitting the phase as factor with 3 levels and group identity as random effect. To conduct all pairwise comparisons between the 3 phases we subsequently used a Tukey post-hoc analysis with adjusted significance level for multiple comparisons. Here too, we used a negative binomial distribution to account for overdispersion of the data.

All analyses were conducted in R Version 4.2 (R Core Team 2022) using the packages lme4 and glmmTMB (Bates et al. 2011; Brooks et al. 2017). All figures were produced from raw, untransformed data.

## RESULTS

### Alloparental care

**Figure 2:**
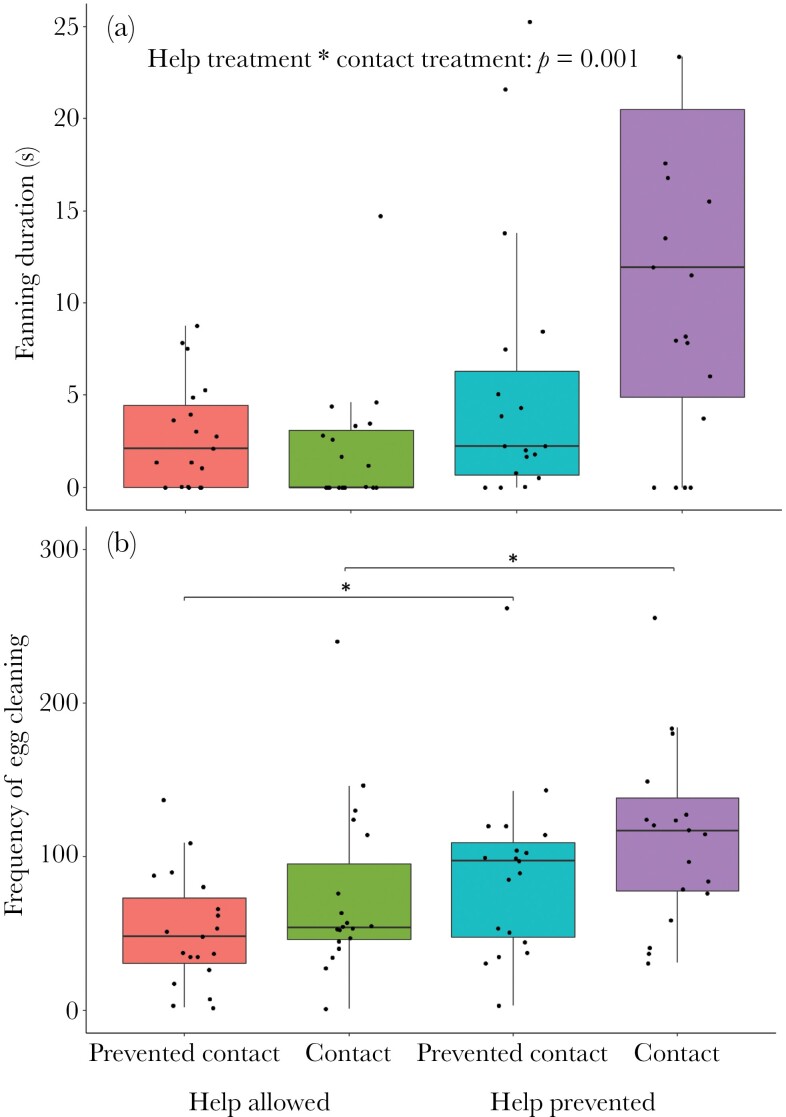
Fanning duration (A) and egg cleaning frequency (B) of helpers in the test phase, as measures of alloparental care in response to the experimental treatments. The boxes depict the median and the interquartile ranges, and the whiskers show the range of the raw data from *N* = 17 observations in each condition. The dots represent data points of each replicate. Stars indicate significance at the level of *p* < 0.05. Full model details are shown in Table 1.

The subordinates provided different amounts of alloparental brood care depending on the behavioral manipulation treatment. The helpers showed more fanning than in all other treatments when they had been prevented from conducting alloparental care in the experience phase and physical contact between breeders and helpers was allowed in the test phase (Table 1, Figure 2A).

**Table 1:** Brood care behavior of the helper in the test phase. Shown are the estimates and incidence rate ratios, 95% confidence intervals, and *p*-values of an LMM and a GLMM modeling the fanning duration and the frequency of egg cleaning during 15 min observations, respectively. As predictors the model included the treatments of the experience and test phases, and their interaction. Both models included the group identity as random effect (*N* = 17 groups, *N* = 68 observations). Significant *p*-values are printed in bold.

Predictors	Fanning			Egg cleaning		
	Estimates	CI	*p*	Incidence Rate Ratios	CI	*p*
(Intercept)	1.02	0.52–1.53	**<0.001**	44.89	30.36–66.37	**<0.001**
Treatment experience phase	0.25	−0.30 to 0.80	0.367	1.7	1.18–2.46	**0.005**
Treatment test phase	-0.39	-0.94–0.16	0.166	1.52	1.05–2.20	**0.027**
Experience * test phase	1.33	0.55–2.11	**0.001**	1	0.63–1.61	0.991
Marginal R^2^/Conditional R^2^	0.234/0.548			0.188/0.625		

Helper egg cleaning in the test phase varied in dependence of both the help prevented in the experience phase and the manipulation of physical contact in the test phase (Table 1, Figure 2B). Help prevented during the experience phase triggered increased levels of egg cleaning behavior of helpers during the test phase. The same effect was produced by the treatment in which dominants could exert overt aggression on the subordinates during the test phase (Figure 2B, Table 1B). In contrast to fanning behavior, the interaction between these two factors was not significant suggesting that this form of energetically cheap alloparental brood care did not only depend on overt aggression from breeders (Table 1B).

### Breeder aggression against the helper

**Figure 3:**
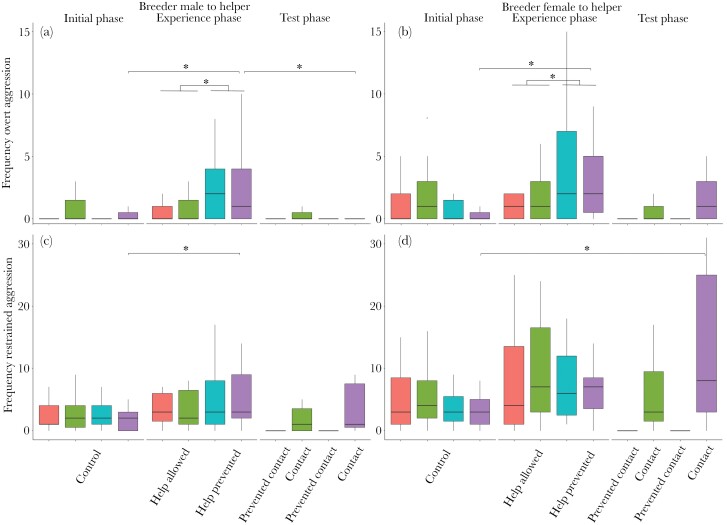
Frequency of overt aggression (A & B) and restrained aggression (C, D) against the helper from the male (A & C) and the female (B & D) breeder during the different phases and treatments. The boxes depict the median and the interquartile ranges, and the whiskers show the range of the raw data from *N* = 17 observations in each condition. Stars indicate significance at the level of *p* < 0.05. Full model details are shown in Tables 2 and 3.

Overt attacks (including physical contact) depended on the treatment and the phase of the experiment. During the experience phase treatment “help prevented”, breeders attacked the helper more often than during the treatment “help allowed” (Figure 3A and B, Table 2A). The longitudinal comparison between the three different phases also suggested an effect of the experimental manipulation “help prevented” during the experience phase on breeder aggression: overt attacks increased from the initial phase to the experience phase (when helping was prevented) and decreased thereafter in the test phase (when helpers resumed brood care, Figure 3A and B, Table 2B). This pattern was the same for both the male and female breeders, with the exception that female breeders did not show a significant decrease of aggression between experience and test phases (Figure 3A and B; Table 2B).

**Table 2:** Overt aggressive behavior of the male and female breeders in A) the experience phase and B) longitudinally throughout the experimental phases when help was prevented during the experience phase and contact was allowed during the test phase. Shown are the incidence rate ratios, 95% confidence intervals, and *p*-values of GLMMs modeling the frequency of male and female aggression during 15 min observations. As predictors the model in A) included the treatment of the experience phase and B) the three different stages of the experiment. Both models included the group identity as random effect (*N* = 17 groups and *N* = 68 or 51 observations, respectively). Significant *p*-values are printed in bold

A)						
Predictors	Male overt aggression			Female overt aggression		
	Incidence Rate Ratios	CI	*p*	Incidence Rate Ratios	CI	*p*
(Intercept)	0.66	0.31 – 1.38	0.271	1.59	0.85 – 2.98	0.147
Help prevented	2.88	1.51 – 5.50	**0.001**	1.93	1.16 – 3.20	**0.011**
Marginal R^2^/Conditional R^2^	0.154/ 0.433			0.068/ 0.438		

B)						
Predictors	Male overt aggression			Female overt aggression		
	Incidence Rate Ratios	CI	p	Incidence Rate Ratios	CI	p
(Intercept)	0.14	0.04 – 0.50	**0.002**	0.77	0.28 – 2.10	0.611
Experience Phase	7.17	3.05 – 16.84	**<0.001**	4.1	1.55 – 10.90	**0.005**
Test Phase	0.83	0.25 – 2.73	0.763	2.37	0.83 – 6.72	0.106

Tukey pairwise comparisons (adjusted for multiple comparisons)						
	Estimate	Standard Error	p	Estimate	Standard Error	p
Initial vs Experience	-1.969	0.436	**<0.001**	-1.412	0.498	**0.018**
Initial vs Test	0.182	0.606	0.951	-0.861	0.533	0.249
Experience vs Test	2.152	0.472	**<0.001**	0.551	0.385	0.335
Marginal R^2^/Conditional R^2^	0.220/0.728			0.191/0.369		

**Table 3:** Restrained aggressive behavior of the male and female breeders in A) the experience phase and B) longitudinally throughout the experimental phases when help was prevented during the experience phase and contact was allowed during the test phase. Shown are the incidence rate ratios, 95% confidence intervals, and *p*-values of GLMMs modeling the frequency of male and female aggression during 15 min observations. As predictors the model in A) included the treatment of the experience phase and B) the three different stages of the experiment. Both models included the group identity as random effect (*N* = 17 groups and *N* = 68 or 51 observations, respectively). Significant *p*-values are printed in bold

A)						
Predictors	Male restrained aggression			Female restrained aggression		
	Incidence Rate Ratios	CI	*p*	Incidence Rate Ratios	CI	*p*
Intercept	6.63	4.13–10.65	**<0.001**	13.61	9.08–20.40	**<0.001**
Help prevented	0.83	0.51–1.37	0.476	0.93	0.54–1.62	0.808
Marginal R^2^/Conditional R^2^	0.006/0.323			0.001/0.092		

B)						
Predictors	Male restrained aggression			Female restrained aggression		
	Incidence Rate Ratios	CI	p	Incidence Rate Ratios	CI	p
(Intercept)	2.3	1.08–4.89	**0.031**	5.71	3.02–10.81	**<0.001**
Experience Phase	2.27	1.18–4.39	**0.015**	1.64	0.86–3.12	0.135
Test Phase	1.94	0.92–4.10	0.083	2.39	1.19–4.79	**0.015**

Tukey pairwise comparisons (adjusted for multiple comparisons)						
	Estimate	Standard Error	p	Estimate	Standard Error	p
Initial vs Experience	−0.82	0.34	**0.048**	−0.5	0.33	0.3
Initial vs Test	−0.66	0.38	0.21	−0.87	0.35	**0.048**
Experience vs Test	0.16	0.32	0.87	−0.38	0.38	0.5
Marginal R^2^/Conditional R^2^	0.090/0.426			0.124/0.219		

Restrained aggression (threat signals) was not equally affected by the treatment, but also changed throughout the phases of the experiment. During the experience phase treatment “help prevented”, breeders showed restrained aggression toward the helper at similar rates as when the helper had the opportunity to conduct brood care (Figure 3C and D, Table 3A). The longitudinal comparison between the three different phases again showed an effect of the manipulation “help prevented” during the experience phase on breeder restrained aggression. Restrained aggression increased from the initial phase to the experience phase in males when helping was prevented (Figure 3C, Table 3B). In females the increase in restrained aggression was significant when the initial phase was compared with the test phase (Figure 3D; Table 3B).

### Helper submission towards breeders

**Figure 4:**
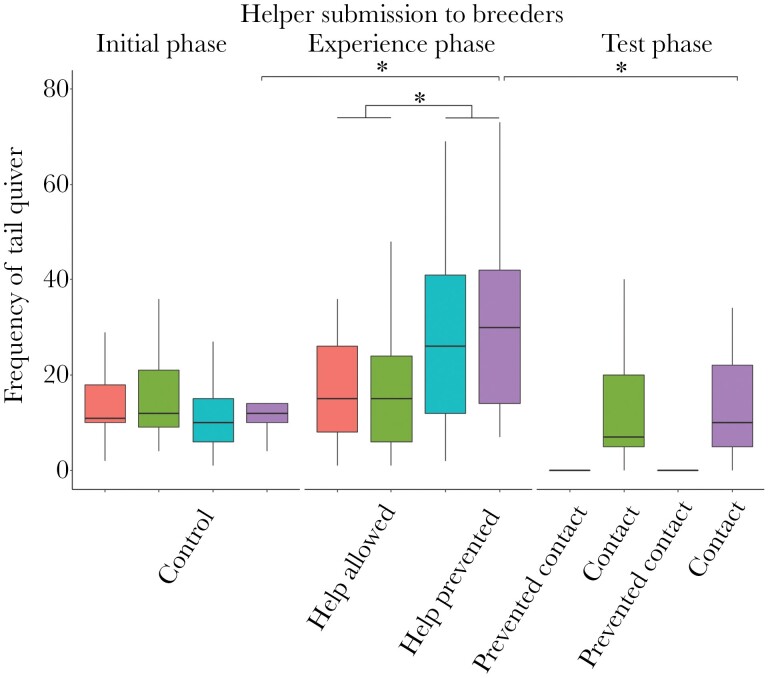
Frequency of tail quivering exhibited by the helper toward the male and female breeders during the different phases and treatments of the experiment. The boxes depict the median and the interquartile ranges, and the whiskers show the range of the raw data from *N* = 17 replicates in each condition. Stars indicate significance at the level of *p* < 0.05. Full model details are shown in Table 4.

Helpers showed more tail quivering towards the breeders in the experience phase treatment “help prevented” than when they were allowed to care for the brood (Figure 4, Table 3A). The longitudinal comparison revealed that the submission rate of the helpers increased from the initial phase to the experience phase (when helping was prevented) and decreased thereafter significantly in the test phase (when helpers reassumed brood care; Figure 4, Table 2B).

**Table 4:** Tail quivering of helper displayed toward the male and female breeders as measure of submission in A) the experience phase and B) longitudinally throughout the experimental phases when help was prevented and contact was allowed. Shown are the incidence rate ratios, 95% confidence intervals, and *p*-values of GLMMs modeling the frequency of male and female aggression during 15 min observations. As predictors, the model in A) included the treatment of the experience phase and in B) the three different stages of the experiment. Both models included the group identity as random effect (*N* = 17 groups and *N* = 68 or 51 observations, respectively). Significant *p*-values are printed in bold

Helper submission			
Predictors	Incidence Rate Ratios	CI	*p*
A)			
(Intercept)	15.72	11.50–21.49	**<0.001**
Help prevented	1.67	1.31–2.11	**<0.001**
Marginal R^2^ / Conditional R^2^	0.119/0.572		
B)			
(Intercept)	14.85	10.46–21.08	**<0.001**
Experience Phase	1.84	1.32–2.58	**<0.001**
Test Phase	0.8	0.53–1.20	0.277

Tukey pairwise comparisons (adjusted for multiple comparisons)			
	Estimate	Standard Error	p
Initial vs Experience	−0.61	0.171	**0.0024**
Initial vs Test	0.224	0.206	0.5266
Experience vs Test	0.834	0.186	**<0.001**
Observations	51		
Marginal R^2^/Conditional R^2^	0.220/0.543		

## DISCUSSION

There are many indications in a wide range of taxa that interacting agents negotiate among each other about the exchange of goods and services (reviewed in Taborsky et al. 2021). Pertinent theoretical studies have outlined the conditions and functionality of such trade and suggest that when social partners negotiate among each other, power asymmetry among them may lead to punishment and enforcement of cooperative behavior (Rubinstein 1982; Clutton-Brock and Parker 1995; McNamara et al. 1999; Gardner and West 2004; McNamara and Leimar 2010; Press and Dyson 2012; Hellmann and Hamilton; 2018). However, the prediction that aggressive behavior can induce cooperation in idle subordinates has rarely been tested. A critical test of this hypothesis must involve experimental manipulations of the responses of both partners to each other’s actions. This was the aim of our study.

Based on the hypothesis that social partners with greater power can enforce service by less powerful partners we predicted that dominant breeders punish idle subordinates, which should in turn enhance their helping effort. We manipulated both the helpful behavior of subordinates and the respective response of the dominants, and we measured the behaviors resulting from these manipulations. Our data reveal that indeed, idle subordinate group members were punished by enhanced overt attacks from dominant breeders, which in turn caused the affected subordinate to increase its helping effort. In contrast, when punishment was experimentally prevented, helpers did not increase energetically costly fanning behavior. This confirms the predictions of punishment and its functionality, which is the basis of negotiations in a pay-to-stay relationship, thereby confirming the causality between social enforcement and helping behavior. Such feedback control and social regulation of cooperation has been suggested to operate also in some other social species, for example in paper wasps (*Polistes fuscatus*; [Reeve and Gamboa 1987]), and we suggest that this may be more widespread than currently assumed.

In asymmetric relationships, the answer to the question how much agents that are more powerful than their social partners can demand from them depends on the alternatives, or “outside options”, of the latter (Reeve 2000; Cant et al. 2012; Cant and Johnstone 2009). In *N. pulcher*, the outside options for subordinate group members are typically poor because of the high mortality risk when leaving a group (Taborsky and Limberger 1980; Taborsky 1984; Heg et al. 2004; Jungwirth et al. 2015). Hence, subordinates should invest as much cooperative effort as is required to be tolerated in a safe territory. A previous experiment investigating the shelter digging effort of helpers, which is an energetically very expensive behavior (Grantner and Taborsky 1998; Taborsky and Grantner 1998), suggested that this is indeed the case. When large, reproductively mature helpers were given the chance to disperse and reproduce elsewhere in a safe environment, they reduced the shelter digging effort in their group (Bergmuller et al. 2005) as predicted by pay-to-stay theory (Kokko et al. 2002). In the field, helpers reduced their cooperative antipredator defense effort shortly before dispersing (Zöttl et al. 2013a). Similarly, in paper wasps, *Polistes dominula*, subordinate group members reduced their cooperative foraging effort in their group when outside options were experimentally enhanced (Grinsted and Field 2017). The extent to which outside options affect the negotiation process between helpers and breeders would be a worthwhile subject for future studies.

In cooperatively breeding animals dominants typically also rely on the presence and cooperation of subordinates. In *N. pulcher*, depending on the local predation pressure breeders may rarely be able to raise young on their own (Brouwer et al. 2005; Groenewoud et al. 2016). In addition, they benefit from the presence of subordinate brood care helpers by load lightening and enhanced productivity (Taborsky 1984; Balshine et al. 2001; Taborsky et al. 2007; Zöttl et al. 2013b). Hence, dominants should not raise the price too high for the tolerance of subordinate group members, but this should depend on the local conditions determining the outside options of subordinates. The remarkable variation of group composition and group size between populations exposed to different levels of predation risk supports this conjecture: in populations with enhanced predation risk the groups contained comparatively more large helpers, which may suggest that the breeders can demand more help from their subordinates when predation pressure is high (Groenewoud et al. 2016). However, our study worked with standardized groups of three individuals and it is currently unclear how group size and composition would affect the results of this experiment.

In contrast to the evidence for PTS, kin selection is apparently of minor importance concerning the relationship among group members in this species, as large helpers are often unrelated to the dominant breeders (Dierkes et al. 2005; Stiver et al.; 2005). In a laboratory experiment, unrelated helpers exhibited higher levels of alloparental brood care than related helpers, confirming an important prediction of PTS and refuting kin selection as an alternative explanation (Zöttl et al. 2013d). Furthermore, only unrelated individuals increased alloparental care when the costs of their presence in the territory were experimentally increased (Zöttl et al. 2013d).

In conclusion, our study demonstrates the causal relationship between punishment for withheld help and subsequent compensation by enhanced cooperation, which reflects an important part of the negotiation process among individuals in asymmetric social relationships.

## SUPPLEMENTARY MATERIAL

Supplementary material can be found at http://www.beheco.oxfordjournals.org/

arac125_suppl_Supplementary_Material

## Data Availability

Analyses reported in this article can be reproduced using the data provided by Zöttl et al 2022.
