# Supplementary material for: Coercion promotes alloparental care in cooperative breeders
Source: Behav Ecol. 2023 Feb 28;34(3):363–72. doi: 10.1093/beheco/arac125 (PMC10183202; doi:10.1093/beheco/arac125)
Supplement: arac125_suppl_Supplementary_Material [file arac125_suppl_supplementary_material.docx]

The introduction of the mesh cage net did not reduce alloparental care levels of the helper (Supplementary figure 1 and supplementary table 1).


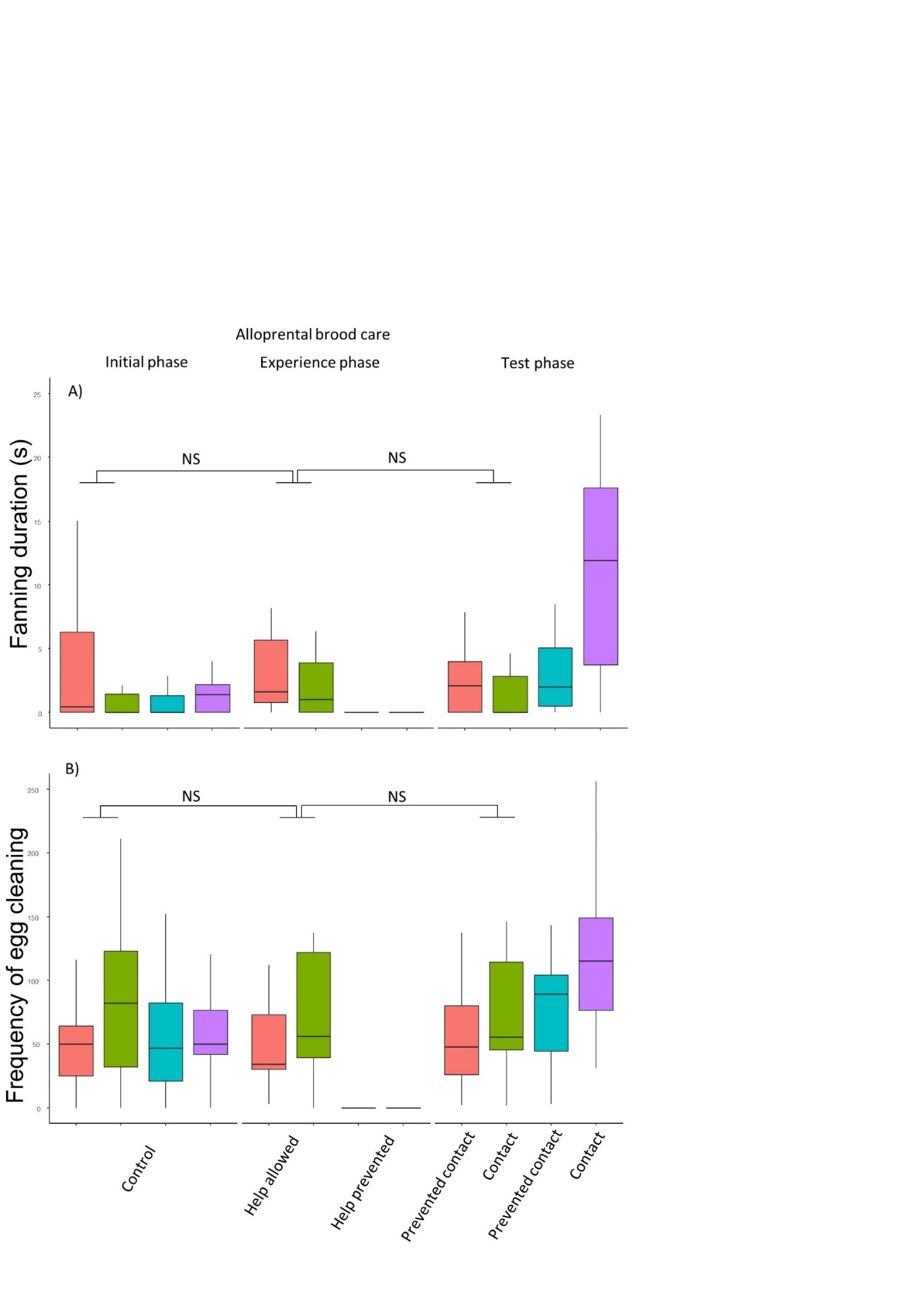


Figure 1: Frequency of A) fanning and B) egg cleaning by the helper during the different phases and treatments The boxes depict the median and the interquartile ranges, and the whiskers show the range of the raw data from N=17 observation in each condition. Full model details are displayed in Supplementary Table 1.

Table 1: Brood care behaviour of the helper in the through all three phases when the experience was “help allowed”. Shown are the estimates and incidence rate ratios, 95% confidence intervals and p-values of a LMM and a GLMM modelling the fanning duration and the frequency of egg cleaning during 15 min observations. As predictors the model included the test phases (factor with 3 levels). Both models included the group identity and whether or not physical contact was possible in the test phase (2 levels) as random effect (N=17 groups, N=104 observations). Significant p-values are printed in bold.

|  |  | **Fanning** | | | **Cleaning** | | |
| --- | --- | --- | --- | --- | --- | --- | --- |
| *Predictors* |  | *Estimates* | *CI* | *p* | *Incidence Rate Ratios* | *CI* | *p* |
| Initial Phase |  | 0.81 | 0.40 – 1.22 | **<0.001** | 56.76 | 36.39 – 88.52 | **<0.001** |
| Experience Phase |  | 0.20 | -0.20 – 0.60 | 0.317 | 0.93 | 0.70 – 1.23 | 0.608 |
| Test Phase |  | 0.02 | -0.38 – 0.41 | 0.929 | 0.98 | 0.75 – 1.30 | 0.909 |
| Marginal R^2^ / Conditional R^2^ |  | 0.009 / 0.247 | | | 0.001 / 0.550 | | |
